# The potential roles of galectin-3 in AKI and CKD

**DOI:** 10.3389/fphys.2023.1090724

**Published:** 2023-02-23

**Authors:** Fengyun Wang, Lixin Zhou, Amity Eliaz, Chang Hu, Xinhua Qiang, Li Ke, Glenn Chertow, Isaac Eliaz, Zhiyong Peng

**Affiliations:** ^1^ Department of Critical Care Medicine, Zhongnan Hospital, Wuhan University, Wuhan, Hubei, China; ^2^ Department of Critical Care Medicine, The First People’s Hospital of Foshan, Foshan, China; ^3^ Department of Medicine, Stanford University School of Medicine, Stanford, CA, United States; ^4^ Amitabha Medical Center, Santa Rosa, CA, United States; ^5^ Center of Critical Care Nephrology, Department of Critical Care Medicine, University of Pittsburgh Medical Center, Pittsburgh, PA, United States

**Keywords:** acute kidney injury, chronic kidney disease, inflammation, galectin-3, renal fibrosis

## Abstract

Acute kidney injury (AKI) is a common condition with high morbidity and mortality, and is associated with the development and progression of chronic kidney disease (CKD). The beta-galactoside binding protein galectin-3 (Gal3), with its proinflammatory and profibrotic properties, has been implicated in the development of both AKI and CKD. Serum Gal3 levels are elevated in patients with AKI and CKD, and elevated Gal3 is associated with progression of CKD. In addition, Gal3 is associated with the incidence of AKI among critically ill patients, and blocking Gal3 in murine models of sepsis and ischemia-reperfusion injury results in significantly lower AKI incidence and mortality. Here we review the role of Gal3 in the pathophysiology of AKI and CKD, as well as the therapeutic potential of targeting Gal3.

## Introduction

Acute kidney injury (AKI) is a prevalent condition with high morbidity and mortality across geographic settings and economic conditions (Hoste et al., 2018). With the rising incidence of AKI, as well as the resultant increase in incidence and progression of chronic kidney disease (CKD), the impact of AKI on long-term health and medical costs likely extends far beyond current estimates.

Galectin-3 (Gal3) is a lectin widely expressed in a variety of organs and tissues, which regulates cell growth, proliferation, differentiation, inflammation, phagocytosis, exocytosis, and fibrosis (Chen and Kuo, 2016; Dong et al., 2018). In addition to the carbohydrate recognition domain (CRD) shared across the galectin family, the chimera-type Gal3 also contains an N-terminal domain that allows the molecule to oligomerize and form pentamers (Rabinovich et al., 2007). Among its many functions, Gal3 recognizes pathogen-associated molecular patterns (PAMPs) and damage-associated molecular patterns (DAMPs) in the cytosol and extracellular space, and can regulate gene transcription in the nucleus (Rabinovich et al., 2007; Dong et al., 2018). Extracellular Gal3 can also bind to cell-surface receptors, modulate cell-cell interactions, and recruit additional lectin molecules to form lattice structures surrounding cells, promoting inflammation and fibrosis (Rabinovich et al., 2007; Dong et al., 2018).

**FIGURE 1:**
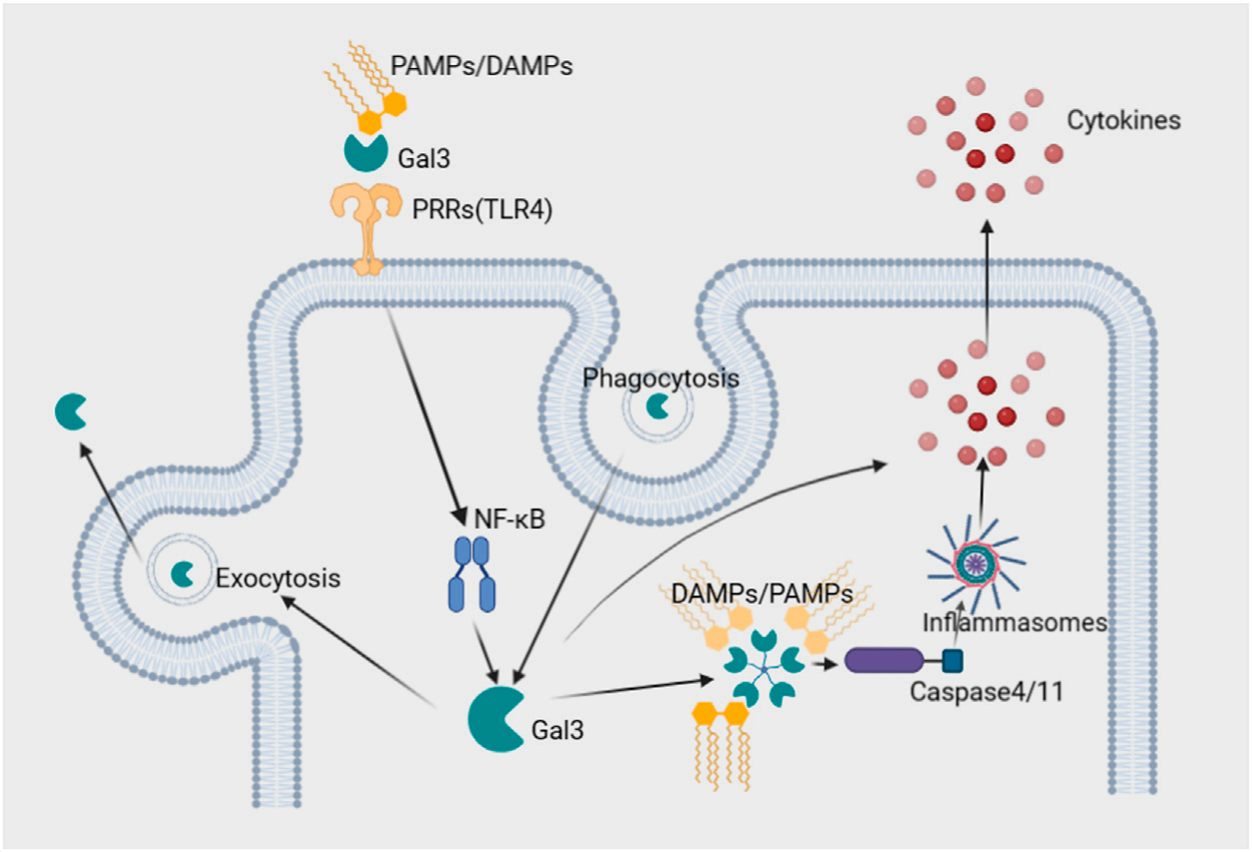
Potential roles of Gal3 in the inflammatory process. 1. Gal3 directly binds cell surface PRRs, such as TLR4, and PAMPs/DAMPs, which form a ternary complex and activate transcription factors such as NF_k_B, amplifying inflammation. 2. PAMPs/DAMPs in the cytosol are recognized by Gal3, which amplifies caspase-4/11 oligomerization and activates noncanonical inflammasomes, facilitating the secretion of cytokines. 3. Recycling of Gal3 mediates phagocytosis and exocytosis, facilitating antigen presentation and promoting inflammation. Abbreviations: DAMPs; damage-associated molecular patterns, Gal3; galectin-3, PAMPs; pathogen-associated molecular patterns, PRRs; pattern recognition receptors, TLR4; toll-like receptor 4.

Gal3 has been implicated in both the activation and amplification of inflammation and fibrosis. It has been shown to mediate endocytosis and exocytosis, likely playing a role in antigen presentation (Lakshminarayan et al., 2014). Cell experiments have demonstrated that Gal3 binds toll-like receptor 4 (TLR4) directly in response to lipopolysaccharide (LPS), promoting and amplifying inflammation (Burguillos et al., 2015). In addition, Gal3 was recently reported to amplify cytosolic caspase-4/11 oligomerization and activation through LPS glycan binding, leading to more intense pyroptosis (Lo et al., 2021). Thus, the interaction between Gal3 and these pattern recognition receptors (PRRs) demonstrates mechanisms by which Gal3 promotes inflammation through cell-surface and intracellular pathways (Burguillos et al., 2015; Lo et al., 2021). Potential mechanisms of Gal3-mediated inflammation are exhibited in Figure 1.

Recent studies have demonstrated that Gal3 may serve as a potential biomarker for various renal and cardiovascular conditions, infections, autoimmune diseases, neurodegenerative disorders, and malignancies (Dong et al., 2018; Hara et al., 2020). In addition, both clinical and basic research have demonstrated the role of Gal3 in the development of AKI (Sun et al., 2021a; Sun et al., 2021b; Boutin et al., 2022). Multiple studies of critically ill patients have shown associations between serum Gal3 concentrations and both AKI and mortality (Sun et al., 2021a; Sun et al., 2021b; Boutin et al., 2022). Further research in animal models has shown that inhibiting Gal3 leads to a significant decrease in the incidence of AKI and mortality in murine models of sepsis and ischemia-reperfusion injury (Sun et al., 2021a; Sun et al., 2021b). These findings suggest that Gal3 may play a critical role in the development and progression of AKI and CKD, and that Gal3 may serve as a potential biomarker in AKI. Here we review recent studies elucidating the role of Gal3 in AKI and CKD, as well as its potential role as a biomarker and therapeutic target.

## Section I: Gal3 and AKI

### Roles in renal ischemia-reperfusion injury (IRI) AKI

Gal3 appears to play an important role in the pathophysiology of renal ischemia-reperfusion injury (IRI) AKI. The mechanism likely involves the production of pro-inflammatory cytokines and the induction of reactive oxygen species (Fernandes Bertocchi et al., 2008). In 2000, Nishiyama *et al.* found that Gal3 mRNA began to increase 2 h after IRI and was augmented by 6.2-fold after 48 h in a rat IRI AKI model utilizing bilateral renal pedicle clamping (Nishiyama et al., 2000). In addition, at 48 h post injury, there was a significant correlation between Gal3 mRNA expression and kidney injury as measured by serum creatinine concentrations (Nishiyama et al., 2000). In a mouse model of IRI utilizing bilateral renal pedicle occlusion, Gal3 knockout mice were found to have less severe acute tubular necrosis (ATN) and enhanced tubular regeneration (Fernandes Bertocchi et al., 2008). Similarly, in a rat IRI AKI model utilizing unilateral renal pedicle clamping, inhibition of Gal3 by modified citrus pectin (MCP) significantly attenuated the rise in creatinine and blood urea nitrogen (BUN), as well as the degree of tubular injury compared to controls (Sun et al., 2021a). Additionally, in a murine model, the overexpression of renal Gal3 correlated with renal inflammation and fibrosis, whereas Gal3 inhibition by MCP attenuated these effects (Martinez-Martinez et al., 2016).

In a study by Dorai et al. (2011) using a warm renal ischemia model, rats treated with a reno-protective cocktail—a mixture of growth factors, mitochondria-protecting biochemicals, and manganese-porphyrin—demonstrated a significant reversal of histopathological changes compared to controls, as well as significantly decreased serum lipocalin-2, mucin-1, and Gal3 concentrations. Cohen et al. (2013) also demonstrated the downregulation of Gal3 by a similar reno-protective cocktail, which was associated with reduced oxidative stress and improved protection of renal parenchymal function following IRI AKI in a rat model. By transplantation of Gal3 wild-type (WT) bone marrow to Gal3 deficient mice and *vice versa*, Prud’homme *et al.* showed that cardiac damage resulting from IRI AKI was Gal3-dependent and was prevented using MCP as a Gal3 inhibitor (Prud’homme et al., 2019). In addition to specific Gal3 inhibitors, the antiplatelet ticagrelor was also shown to exert a reno-protective effect by inhibiting Gal3 and caspase-3 activity in a mouse IRI model (Mansour et al., 2022).

### Roles in cisplatin-induced AKI

A study by Li et al. (2018) revealed that overexpression of Gal3 induced cell cycle arrest and apoptosis in renal cells, decreasing their viability, whereas inhibition of Gal3 by MCP significantly attenuated the toxic proapoptotic effects of cisplatin. In addition, MCP-treated mice exhibited improved kidney function and decreased renal fibrosis compared to controls after cisplatin-induced AKI (Li et al., 2018). In contrast, a study by Volarevic et al. (2019) reported that cisplatin-induced apoptosis was exacerbated in Gal3 knockout mice and WT mice that received a Gal3 inhibitor, while recombinant Gal3 blunted apoptosis in Gal3 deficient mice with cisplatin-induced AKI. Additionally, in a recent study by Al-Salam et al. (2021), cisplatin treatment in Gal3 knock-out mice was associated with a greater burden of ATN, as well as increased levels of plasma urea, creatinine, cathepsin B, and cathepsin D compared to WT mice. Due to the limited evidence and variable results specific to cisplatin-induced AKI, the specific role of Gal3 in the pathophysiology of cisplatin-induced AKI requires further investigation. However, the selective effects of Gal3 depletion or inhibition *versus* Gal3 knockout, as well as the types of inhibitors used, may contribute to these paradoxical findings.

### Roles in folic-acid-induced AKI

In a rat model of folic-acid-induced AKI, Gal3 mRNA was upregulated in kidney tissue at 2 h after injury, and increased levels were maintained for at least 7 days post-injury (Nishiyama et al., 2000). In another study of folic-acid-induced AKI, Gal3 expression was elevated in injured tubules after folic acid administration, and Gal3 inhibition reduced proinflammatory cytokines, renal fibrosis, and apoptosis at 2 weeks post-injury (Kolatsi-Joannou et al., 2011). These findings suggest that inhibition or depletion of Gal3 may be protective in folic acid-induced AKI (Kolatsi-Joannou et al., 2011).

### Roles in infection-associated AKI

Evidence from multiple studies demonstrates that myeloid cell-derived Gal3 drives acute and chronic inflammation, including infection-mediated inflammation, and warrants further evaluation of Gal3 as a therapeutic target (Ferreira et al., 2018; Wang et al., 2019; Chiu et al., 2020; Guo et al., 2021; Humphries et al., 2021). In a clinical cohort study, median Gal3 concentrations were elevated 1.4-fold in pneumonia and 2.7-fold in sepsis (Mueller et al., 2015). Additionally, in our previous work, we demonstrated that serum Gal3 concentrations predicted AKI in patients with sepsis (Sun et al., 2021b). In a study by Ferrer *et al.*, macrophage depletion and Gal3 knockout were associated with greater bacterial burden and worsened subacute nephritis compared to WT mice in a mouse model of leptospirosis (Ferrer et al., 2018). In addition, in a murine model of sepsis using cecal ligation and puncture, inhibition of Gal3 significantly reduced AKI incidence and mortality (Sun et al., 2021b). Thus, Gal3 depletion or inhibition may serve as a therapeutic approach in sepsis and sepsis-associated AKI.

## Section II: Gal3 and CKD

### CKD progression

Multiple studies have demonstrated an association between elevated serum Gal3 concentrations and accelerated progression of CKD following AKI. Among 352 patients with CKD, serum Gal3 concentration directly correlated with serum creatinine (Cr) level and the urine protein-to-Cr ratio (Kim et al., 2021). Additionally, in a combined analysis of two clinical studies, elevated serum Gal3 concentration was associated with poor clinical outcomes in participants with impaired kidney function, but not in participants with normal or near-normal kidney function (Drechsler et al., 2015). Mean Gal3 concentrations were higher with poorer kidney function—12.8 ± 4.0 ng/ml for estimated glomerular filtration rate (eGFR)≥90 ml/min, 15.6 ± 5.4 ng/ml for eGFR 60–89 ml/min, 23.1 ± 9.9 ng/ml for eGFR<60 ml/min, and 54.1 ± 19.6 ng/ml for patients requiring dialysis—and higher serum Gal3 concentrations were associated with cardiovascular events, infection, and all-cause mortality in patients with impaired kidney function (Drechsler et al., 2015). Among a cohort of 2,450 participants in the Framingham Offspring Study, elevated plasma concentrations of Gal3 were associated with increased odds of decline in eGFR and of incident CKD (O'Seaghdha et al., 2013). Additionally, in a study evaluating a community-based population of 9,148 patients without previous CKD or heart failure, higher plasma Gal3 concentrations were associated with an increased risk of incident CKD, most notably among those with hypertension (Rebholz et al., 2018).

### Mortality in CKD

Several studies suggest that Gal3 concentrations are associated with mortality in patients with CKD (Drechsler et al., 2015; Alam et al., 2019). In a study evaluating 150 patients with CKD, the discrimination of serum Gal3 concentrations for mortality based on the area under the receiver operating characteristic curve (AUC-ROC) was higher than that of serum cystatin C and serum creatinine (AUC-ROC: Gal3 = 0.89, cystatin C = 0.83; creatinine = 0.85) (Ji et al., 2017). The 6-year kidney survival rates of the low Gal3 group and high Gal3 group were 47.3% and 22.8% respectively (*p* < 0.01) (Ji et al., 2017). In addition, an observational cohort of 883 patients with CKD showed that higher serum Gal3, GDF-15, and sST2 concentrations were associated with a greater likelihood of death (Tuegel et al., 2018). Another study using an immunohistochemical technique demonstrated that higher glomerular and extraglomerular Gal3 immunoreactivity was associated with a lack of response to steroids in children with diffuse mesangial proliferation and focal segmental glomerulosclerosis (Ostalska-Nowicka et al., 2009).

### Cardiovascular events in CKD and ESRD

Multiple studies have exhibited an association between Gal3 and cardiovascular events in patients living with CKD and end-stage renal disease (ESRD). A study of 163 patients with CKD and 105 controls demonstrated an association between serum Gal3 and both brain natriuretic protein (BNP) and high sensitivity troponin in patients with CKD (Chan et al., 2020). In another study, measures of Gal3, pentraxin-3, MMP-9, and eGFR in combination predicted higher or lower risks of cardiovascular events (Miljkovic et al., 2017). Similarly, in patients receiving hemodialysis, elevated Gal3 concentration was associated with higher rates of cardiovascular death (Salib et al., 2021). An additional study of patients receiving hemodialysis found that arteriovenous fistula stenosis was associated with the expression of Gal3 (Ruan et al., 2021). Furthermore, there was a positive correlation of serum Gal3 with neointima development (Ruan et al., 2021). In a prospective cohort study, higher serum Gal3 concentration was associated with a higher risk of cardiovascular mortality in patients receiving maintenance hemodialysis (hazard ratio (HR) = 2.13, 95% CI 1.07–4.26) (Liu et al., 2022). In addition, a cohort study of 2,477 participants showed that greater longitudinal increase in plasma Gal3 concentration was associated with increased incidence of heart failure and all-cause mortality (Ghorbani et al., 2018). In a study of 130 patients with CKD, serum Gal3 concentrations were directly correlated with C-reactive protein (CRP) concentrations and inversely correlated with eGFR, while higher serum Gal3 and CRP concentrations were associated with vascular reactivity index, a measure of endothelial dysfunction (Hsu et al., 2021).

## Section III: Gal3 and other kidney diseases

### Kidney transplant (KT)

Recent human and animal studies have found an association between Gal3 concentrations and kidney transplant outcomes. An analysis of 561 kidney transplant recipients found that serum Gal3 concentrations were elevated and were independently associated with the risk of late graft failure (Henderson et al., 2008). Additionally, in a murine model of chronic allograft injury, Dang et al. (2012) found that Gal3 null mice had significantly improved preservation of renal tubules and reduced interstitial fibrosis following kidney transplant compared to controls (Dang et al., 2012). Interestingly, a study comparing patients receiving hemodialysis or transplantation, found that patients who underwent kidney transplant had significantly lower Gal-3 concentrations at 3 months post-operatively, while patients that continued hemodialysis did not have significantly different Gal-3 concentrations (Tan et al., 2014). Given these findings, Gal3-targeted therapies warrant further investigation in the prevention of tubulointerstitial fibrosis following transplant.

### Autoimmune nephropathy

Given its critical role in modulation of the immune response, inflammation, and fibrosis, Gal3 has been implicated in the pathophysiology of autoimmune nephropathies (Saccon et al., 2017). In an analysis of kidney tissue from patients with lupus nephritis, LGALS3—the interferon-regulated gene that encodes Gal3—was directly correlated with disease activity (Almaani et al., 2019). Furthermore, patients that achieved complete response demonstrated a lower abundance of LGALS3 (Almaani et al., 2019). Another study found that both glomerular Gal3 expression and serum Gal3 concentrations were elevated in patients with systemic lupus erythematosus (SLE) compared to controls (Kang et al., 2009). In addition, Gal-3 expression was found to correlate directly with anti-dsDNA and inversely with complement 3 and 4 levels (Kang et al., 2009).

### Diabetic nephropathy

Gal3 has been implicated in the progression of diabetic nephropathy. In addition to its role in inflammation and fibrosis, Gal3 can bind directly to the insulin receptor and inhibit downstream signaling, thus decreasing insulin sensitivity (Li et al., 2016). In a murine experimental model of diabetes, Gal3 knockout, Gal3 heterozygous depletion, and Gal3 inhibition resulted in increased insulin sensitivity and glucose tolerance compared to controls [72]. In a prospective study of patients with diabetic nephropathy, mean serum concentrations of Gal3 were significantly higher in patients with macroalbuminuria compared to patients with microalbuminuria or without albuminuria (Hodeib et al., 2019). In another study of 1,320 patients with type 2 diabetes and an eGFR of 30 mL/min/1.73 m^2^ or higher, elevated serum Gal3 concentrations were associated with progressive kidney disease (Tan et al., 2018). Similarly, in a study of patients with type 1 diabetes, serum Gal3 was elevated and associated with lower eGFR, as well as higher urine albumin to creatinine ratio (de Boer et al., 2017).

### Hypertensive nephropathy

In patients with hypertension, higher serum Gal3 concentrations were inversely correlated with eGFR (Lau et al., 2021). In a study of 107 patients with hypertension, serum Gal3 was associated with increased odds of left ventricular remodeling before and after adjusting for body mass index (BMI) and systolic blood pressure (SBP) (OR: 14.76; 95% CI, 5.39–27.76, *p* < 0.001) (Yao et al., 2016). A preclinical study of spontaneously hypertensive rats found that MCP-mediated Gal3 inhibition attenuated early kidney damage independent of blood pressure levels, as evidenced by reduced albuminuria and improved kidney function, as well as decreased interstitial fibrosis, epithelial-mesenchymal transition, and inflammation on kidney biopsy (Martinez-Martinez et al., 2018). Frenay et al. (2015) also demonstrated that inhibition of Gal3 attenuated hypertensive nephropathy in rats, as evident by improved kidney function, reduced proteinuria, and decreased structural kidney damage (Frenay et al., 2015). In another murine hypertension model, Gal3 inhibition was observed to attenuate renal inflammation and fibrosis in experimental hyperaldosteronism, independent of blood pressure (Martinez-Martinez et al., 2015).

### Renal fibrosis

Recent studies have demonstrated critical roles for Gal3 in fibrogenesis affecting multiple organ systems, including the liver, kidney, lung, and myocardium (Li et al., 2014). In a study of 249 patients with CKD who underwent kidney biopsy, plasma Gal3 concentrations were directly correlated with interstitial fibrosis and tubular atrophy, and were inversely correlated with eGFR (*p* = 0.005) (Ou et al., 2021). Similarly, in a prospective study of 280 patients who underwent kidney biopsies, higher urinary Gal3 concentrations were associated with more severe interstitial fibrosis (Ou et al., 2022).

Murine models have also demonstrated associations between Gal3 and renal fibrosis. In a murine model of unilateral ureteral obstruction (UUO), Gal3 expression in the renal interstitium and tubular epithelium was significantly higher than Gal3 expression in controls (Henderson et al., 2008). Additionally, Gal3 knockout mice, as well as macrophage ablation, resulted in significantly reduced renal fibrosis, and the adoptive transfer of WT Gal3-positive macrophages restored the fibrotic phenotype in Gal3 knockout mice (Henderson et al., 2008). The authors concluded that Gal3 secretion by macrophages plays a critical role in the mechanism linking macrophages to renal fibrosis (Henderson et al., 2008). Similarly, in experimental hyperaldosteronism, cardiac and renal fibrosis was associated with the expression of Gal3, which was prevented by Gal3 inhibitor MCP or genetic deletion of Gal3 (Calvier et al., 2015). In another UUO mouse model, Twist1 deficient macrophages demonstrated significantly decreased renal interstitial fibrosis 14 days after UUO, and Gal3 expression was significantly reduced in Twist1 deficient macrophages compared to controls (Wu et al., 2022). Additionally, Twist1 was shown to directly activate Gal3 transcription, and Gal3 upregulation recovered Twist1-mediated M2 macrophage polarization in Twist1 deficient macrophages, suggesting that Twist1/Gal3 signaling modulates macrophage plasticity and promotes renal fibrosis (Wu et al., 2022). In rat models of obesity and aortic stenosis, Gal3 expression paralleled the degree of interstitial fibrosis, and inhibition of Gal-3 with MCP normalized Gal3 levels and prevented the progression of renal fibrosis (Martinez-Martinez et al., 2016).

## Section IV: The therapeutic potential of targeting Gal3

Gal3 is a carbohydrate-binding lectin implicated in the pathophysiology of a wide array of inflammatory and fibrotic conditions, including AKI and CKD. Gal3 concentrations are elevated in AKI and CKD, and Gal3 inhibitors have been shown to downregulate inflammation and fibrosis in a variety of diseases (Supplementary Table S1) (Drechsler et al., 2015; Ji et al., 2017; Alam et al., 2019; Sun et al., 2021b; Kim et al., 2021; Boutin et al., 2022). In murine models, Gal3 inhibitors have been shown to significantly decrease kidney injury in a variety of conditions, including sepsis-associated AKI, renal fibrosis in experimental hyperaldosteronism, hypertensive nephropathy, interstitial fibrosis following transplant, and renal fibrosis in IRI AKI. Given these findings, Gal3 inhibition or depletion may serve as a potential therapeutic target in AKI and CKD and warrants further investigation.

## Conclusion

As demonstrated in the current literature, Gal3 appears to play a key role in the pathophysiology of AKI and CKD. Gal3 is associated with the development and progression of various etiologies of AKI, and inhibition of Gal3 results in improved kidney function, as well as decreased inflammation and fibrosis. Current evidence suggests that Gal3 may serve as a potential biomarker and therapeutic target in AKI and CKD, and warrants further investigation.

## Supplementary material

SUPPLEMENTARY TABLE S1 Serum and plasma Gal3 levels in AKI and CKD by severity. Mean and median Gal3 concentrations in patients with lower severity of disease and controls compared to higher severity of disease. Abbreviations: AKI, acute kidney injury; CKD, chronic kidney disease; eGFR, estimated glomerular filtration rate; Gal3, galectin-3.

## Abbreviations

AKI, Acute kidney injury; CKD, Chronic kidney disease; Gal3, galectin-3; MCP, Modified citrus pectin; DAMPs, Damage-associated molecular patterns; PAMPs, Pathogen-associated molecular patterns; PRRs, Pattern recognition receptors; CVD, cardiovascular diseases; IRI, ischemia-reperfusion injury; ATN, Acute tubular necrosis; GFR, Glomerular filtration rate; ESRD, End-stage renal disease.
