# Supplementary material for: The potential roles of galectin-3 in AKI and CKD
Source: Front Physiol. 2023 Feb 23;14:1090724. doi: 10.3389/fphys.2023.1090724 (PMC9995706; doi:10.3389/fphys.2023.1090724)
Supplement: Supplementary file 1 [file Table1.docx]

Supplementary Table 1: Serum and plasma Gal3 levels in AKI and CKD by severity.

***Lower Severity and Controls Higher Severity***

|  | Condition | Severity | Serum or Plasma Gal3 (ng/ml) | Severity | Serum or Plasma Gal3 (ng/ml) |
| --- | --- | --- | --- | --- | --- |
| Boutin, L. (2022) | AKI | Stage 1 AKI | 23.6 [18.2, 34.2] | Stage 3 AKI | 38.0 [24.5, 57.1] |
| Sun, H. (2021) | Sepsis | Sepsis without AKI | 5.3±0.5 | Sepsis-associated AKI | 11.2±1.6 |
| Alam, M. L. (2018) | CKD | eGFR 65ml/min per 1.73 m^2^ | <9.04 | eGFR 39ml/min per 1.73 m^2^ | >16.76 |
| Kim, A. J. (2021) | CKD | CKD without progression | 9.332±6.083 | CKD with ≥50% eGFR reduction | 13.051±10.196 |
| Ji, F.  (2016) | CKD | Controls (eGFR 102.2±26.2 ml/min per 1.73 m^2^) | 4.2±1.2 | CKD (eGFR 46.0±28.4 ml/min per 1.73 m^2^) | 6.0±0.9 |
| Drechsler, C. (2015) | CKD | Controls (eGFR≥90 ml/min per 1.73 m^2^ ) | 12.8±4.0 | CKD (eGFR<60 ml/min per 1.73 m^2^) | 23.1±9.9 |

Serum and plasma Gal3 levels in AKI and CKD by severity. Mean and median Gal3 concentrations in patients with lower severity of disease and controls compared to higher severity of disease. Abbreviations: AKI: acute kidney injury; CKD: chronic kidney disease; eGFR: estimated glomerular filtration rate; Gal3: galectin-3.
